# Quadruple Pregnancy

**Published:** 1885-08

**Authors:** M. Arthur

**Affiliations:** Waverly, Codington County, Dakota


QUADRUPLE PREGNANCY. *By* M. ARTHUR, M. D., *Waverly, Codington County, Dakota.*

I take pleasure in reporting a case of quadruple pregnancy that has terminated happily.

At six A. M., July 3rd, I was called to see Mrs. D. R. Barnett. I found her greatly enlarged, with extreme œdema of the feet and legs. Labor had commenced four hours before Pains were frequent and regular, the os dilatable, with a vertex presenting, liquor *amnii* had not passed. After rupturing the sac, labor progressed more rapidly, and about eight o'clock a female child was born. The pains continuing, I examined again and discovered a breech presenting also in the unbroken sac; shortly after rupturing this a male babe was delivered. Much to my surprise the pains continued, and after caring for the second child I examined again, finding another vertex engaged and also in its own sac. This I could not rupture in the usual manner, with my finger, but had to use a pair of blunt scissors. In fifteen or twenty minutes, a second female babe was safely delivered. I thought surely this was enough, but no, the pains continued constant and strong. Thinking it must be the placenta, I cared for the third babe before examining. When I was ready, I discovered a second breech presentation, this also in its own sac. This sac I had to rupture as the previous one, with the scissors, on account of the toughness of the membrane. In about twenty minutes the second male, or fourth child, was delivered, but apparently dead and bloodless. However, after working over it for about ten minutes, respiration and circulation were well established. I delivered the placenta in fifteen or twenty minutes after the last child; it was in one piece and very large, although the union of four placentæ could be distinctly seen with their four cords attached.

The babes weighed in the afternoon of the same day twenty-one and a half pounds in all. The girls 5¼ pounds each and the boys 5½ pounds each. The mother and children are all progressing finely at the present writing, the babes being nursed on the mixed plan.

The parents are Scotch, coming to this country three years ago. They have five children previous to the late addition, one of which is Dakota born. The lady is 35 or 36 years of age, medium size and has been robust.

